# Food-Derived Antioxidant Polysaccharides and Their Pharmacological Potential in Neurodegenerative Diseases

**DOI:** 10.3390/nu9070778

**Published:** 2017-07-19

**Authors:** Haifeng Li, Fei Ding, Lingyun Xiao, Ruona Shi, Hongyu Wang, Wenjing Han, Zebo Huang

**Affiliations:** 1 Center for Bioresources & Drug Discovery and School of Biosciences & Biopharmaceutics, Guangdong Pharmaceutical University, Guangzhou 510006, China; lihf@gdpu.edu.cn (H.L.); 15692435199@163.com (F.D.); 15521217084@163.com (R.S.); 15626200071@163.com (H.W.); 15622104553@163.com (W.H.); 2 School of Pharmaceutical Sciences, Wuhan University, Wuhan 430071, China; Mandy.Xiao@infinitus-int.com

**Keywords:** polysaccharide, antioxidant, oxidative stress, inflammatory stress, proteotoxic stress, neurodegeneration

## Abstract

Oxidative stress is known to impair architecture and function of cells, which may lead to various chronic diseases, and therefore therapeutic and nutritional interventions to reduce oxidative damages represent a viable strategy in the amelioration of oxidative stress-related disorders, including neurodegenerative diseases. Over the past decade, a variety of natural polysaccharides from functional and medicinal foods have attracted great interest due to their antioxidant functions such as scavenging free radicals and reducing oxidative damages. Interestingly, these antioxidant polysaccharides are also found to attenuate neuronal damages and alleviate cognitive and motor decline in a range of neurodegenerative models. It has recently been established that the neuroprotective mechanisms of polysaccharides are related to oxidative stress-related pathways, including mitochondrial function, antioxidant defense system and pathogenic protein aggregation. Here, we first summarize the current status of antioxidant function of food-derived polysaccharides and then attempt to appraise their anti-neurodegeneration activities.

## 1. Introduction

Oxygen is essential for normal life of aerobic organisms. Due to its high redox potential, oxygen is inevitably involved in the production of reactive oxygen species (ROS) such as superoxide anion, hydroxyl radical and hydrogen peroxide. ROS are known to play an important role in a variety of cellular functions including signal transduction and regulation of enzyme activity [1,2]. Excessive ROS, on the other hand, can also interact with biological molecules and generate by-products such as peroxides and aldehydes, which can cause damages to architecture and function of cells [3,4]. Under normal circumstances, cells have a set of antioxidant defense system, including enzymatic antioxidants such as superoxide dismutase (SOD), catalase (CAT) and glutathione peroxidase (GPx) and non-enzymatic antioxidants such as glutathione and vitamins, to combat excessive ROS [5,6]. However, when a detrimental stress compromises the antioxidant defense system, a fraction of ROS may escape the intrinsic clearance machinery and induce a state of oxidative stress, leading to cell dysfunction [7,8].

Growing evidence has demonstrated that oxidative stress is implicated in the development and progression of many chronic diseases such as neurodegenerative diseases (NDD) and diabetes [9,10,11]. NDD, including Alzheimer’s disease (AD), Parkinson’s disease (PD) and Huntington’s disease (HD), are a group of chronic disorders pathologically characterized by selective and progressive loss of neurons [12]. Clinical evidence has shown that NDD patients display an oxidative stress-related manifestation, including increases of ROS level, lipid peroxidation and protein oxidation [13,14]. Recent studies have revealed that ROS-induced peroxidation products, such as the lipid peroxidation product malondialdehyde (MDA) and the protein oxidation product carbonyl groups, can damage other cellular components and exacerbate neuronal dysfunction, further demonstrating the detrimental consequence of oxidative stress in neurodegeneration [4,15,16]. Therefore, strategies to reduce oxidative damages are shown to be beneficial to alleviate neurodegenerative symptoms. For example, intake of foods rich in antioxidant ingredients has shown potentials to prevent oxidative stress-related conditions, including NDD [17,18,19]. Among the reported ingredients, polysaccharides, an important type of natural polymers consisting of monosaccharide units that contain multiple free hydroxyl groups, are shown to have both in vitro and in vivo antioxidant activities [20,21]. Here, we first review the antioxidant effects of food-derived polysaccharides and then focus on their protective function against neurodegeneration.

## 2. Reduction of Oxidative Stress by Food-Derived Polysaccharides

During the last decade, a large body of evidence has shown that polysaccharides and glycoconjugates from a variety of natural sources, including bacteria, fungi, algae, plants and animals, have antioxidant potentials [20,21,22,23]. In particular, polysaccharides isolated from functional and medicinal foods as well as from common foods have drawn great attention in antioxidant studies. Here, we attempt to summarize recent studies of antioxidant polysaccharides from food resources, including vegetables, fruits, cereals, beans, mushrooms, tea, milk products and meat (Table 1) [24,25,26,27,28,29,30,31,32,33,34,35,36,37,38,39,40,41,42,43,44,45,46,47,48,49,50,51,52,53,54,55,56,57,58,59,60,61,62,63,64,65,66,67,68,69,70,71,72,73].

### 2.1. Reduction of Free Radical and Peroxidation Product Levels

Many food-derived polysaccharides are reported to have potent reducing power and free radical scavenging ability in vitro. For example, we have previously isolated a polysaccharide from *Nostoc commune*, a widespread microalga with a long history as food and medicine, and found that the polysaccharide is capable of scavenging both superoxide anion and hydroxyl radicals in vitro [66]. The antioxidant capability of polysaccharides is shown to be related with their functional groups such as hydroxyl, amino, carbonyl and carboxyl groups, e.g., the scavenging capacity of chitosan against superoxide radicals is correlated with its number of hydroxyl and amino groups [74]. The polysaccharide fractions from *Zizyphus jujuba* with higher uronic acid content exhibit stronger free radical scavenging activities than other polysaccharide fractions from the same species containing no uronic acid [75]. These functional groups in polysaccharides can donate hydrogen to electron-deficient free radicals to generate alkoxyl products, which accelerate intramolecular hydrogen abstraction and further induce spirocyclization reaction to prevent radical chain reaction [22,76]. Interestingly, free radicals are usually generated via transition metal ions in in vitro antioxidant assays. In Fenton reaction, for instance, ferrous ion is used to catalyze superoxide or hydrogen peroxide to generate hydroxyl radicals [77]. Therefore, the direct scavenging effect of polysaccharides against free radicals may also be through chelating ions. For example, the polysaccharide fraction GAPS-1 isolated from *Aloe barbadensis* has a higher chelating ability against ferrous ion and meanwhile exhibits stronger scavenging effect against hydroxyl radicals as compared to SAPS-1, another polysaccharide fraction isolated from the same species [51]. Moreover, monosaccharide composition and substitution groups of polysaccharides are reported to play important roles in their chelating capacity, e.g., the chelating ability of the polysaccharides from *Zizyphus jujuba*, a well-known traditional food, against ferrous ion is positively correlated with their galacturonic acid contents [50].

In addition to scavenge free radicals in vitro, antioxidant polysaccharides are also shown to reduce the levels of ROS and associated peroxidation products in cellular and animal models under oxidative stress. For instance, a polysaccharide from the common fungus *Auricularia auricular* is capable of increasing the survival rate and reducing the ROS level in hydrogen peroxide-stressed *Caenorhabditis elegans* [59], while the wheat bran-derived feruloyl oligosaccharides can reduce MDA content and suppress protein carbonyl formation in human erythrocytes exposed to 2,2′-Azobis(2-amidinopropane) dihydrochloride, a potent free radical generator [34]. It is well established that peroxidation products can modify cellular components, leading to cell damages. For instance, MDA interacts with proteins and DNA to generate covalent adducts with mutagenic and carcinogenic effects [3], while protein carbonyl groups can cause rapid degradation of proteins [78]. Therefore, reduction of peroxidation product contents may contribute to the protective effects of feruloyl oligosaccharides against oxidative stress.

Mitochondria are the main source of ROS and energy production in cells. However, mitochondrial dysfunction, including mitochondrial membrane potential (MMP) decline, respiratory chain malfunction and calcium dysregulation, can accelerate ROS generation and reduce ATP generation, leading to oxidative damage and energy deficiency [79,80]. In a vicious cycle, excessive ROS further impair mitochondrial components such as membrane lipids and DNA, resulting in a secondary mitochondrial dysfunction that amplifies oxidative stress [81,82]. Therefore, restoring mitochondrial function is a beneficial strategy to reduce oxidative impairment. Interestingly, recent reports have revealed that the antioxidant function of food-derived polysaccharides is associated with the alleviation of mitochondrial dysfunction. For example, we have recently shown that the polysaccharide DiPS from *Dictyophora indusiata*, an edible mushroom traditionally used for inflammatory and neural diseases, can reduce paraquat-mediated increase of ROS level through elevating MMP in *C. elegans* [58]. A polysaccharide from *Ganoderma lucidum*, a well-known mushroom traditionally used to delay ageing and enhance immune function, is able to attenuate isoproterenol-induced cardiotoxicity via increasing MMP and mitochondrial complex activity in rats [56]. In addition to mitochondria, several other biochemical pathways such as NADPH oxidase also contribute to ROS production [9]. Interestingly, a recent study has found that a polysaccharide from *Sophora subprosrate*, a medicinal food used for inflammatory disorders, can reduce superoxide anion in porcine circovirus type 2-infected murine macrophage RAW264.7 cells by inhibiting the expression of NADPH oxidase, which is a major enzyme responsible for generating superoxide anion in phagocytes [43]. Together, these findings demonstrate that antioxidant polysaccharides can inhibit cellular ROS generation through multiple pathways.

### 2.2. Improvement of the Antioxidant Defense System

A number of studies have revealed that food-derived polysaccharides can reduce oxidative stress and associated damages through modulation of antioxidant enzymes in experimental models. For example, we have recently found that the acidic polysaccharide EbPS-A1 from *Epimedium brevicornum*, a functional food used for a variety of medical conditions including neurological disorders, can increase oxidative survival and reduce ROS level and MDA content of both wild-type and polyglutamine (polyQ) *C. elegans* under paraquat-induced oxidative stress. The protective effect of EbPS-A1 against paraquat toxicity is shown to be related with increasing SOD and CAT activities [38]. Interestingly, the polysaccharides isolated from the tonic food *Chuanminshen violaceum* are also shown to up-regulate the mRNA expression levels of SOD isoforms and CAT and enhance the activities of these antioxidant enzymes in mice injected with d-galactose [39], an ageing-promoting agent that induces cognitive and motor performance deterioration similar to AD symptoms via oxidative stress and mitochondrial dysfunction [83].

In addition to their effect on antioxidant enzymes, several food-derived polysaccharides are also reported to have modulatory function on non-enzyme components of the cellular antioxidant system. For example, a polysaccharide from *Anoectochilus roxburghii*, a medicinal food used to treat a variety of chronic diseases such as hepatitis and diabetes, is shown to attenuate oxidative stress by increasing glutathione level as well as antioxidant enzyme activities in the hepatic tissue of mice injected with carbon tetrachloride, an organic chemical that can induce hepatotoxicity through increased oxidative stress [84]. Interestingly, *A. roxburghii* polysaccharide is also shown to reduce the mRNA levels of inflammation-related genes including tumor necrosis factor alpha (TNF-α) and interleukin-6 (IL-6) [52]. Oxidative stress is known to increase the expression of TNF-α, a key cytokine that promotes inflammation, while elevated TNF-α level can activate NADPH oxidase, ultimately leading to ROS overproduction [85,86].

### 2.3. Regulation of Oxidative Stress-Related Signaling

A number of signaling pathways, such as those involving nuclear factor erythroid 2-related factor 2/antioxidant response element (Nrf2/ARE), mitogen-activated protein kinases (MAPKs), phosphoinositide 3 kinase/Akt (PI3K/Akt) and insulin/insulin-like growth factor-1 signaling (IIS), are known to be associated with cellular responses to multiple stresses including oxidative stress [87,88,89]. For instance, Nrf2, a basic region leucine-zipper transcription factor, plays an important role in cellular antioxidant response. When Nrf2 is activated, it translocates into nucleus and binds to ARE, leading to up-regulation of genes involved in cellular antioxidant and anti-inflammatory defense as well as mitochondrial protection [87]. Interestingly, some food-derived polysaccharides are recently reported to exert their antioxidant activity via Nrf2/ARE pathway in cellular and animal models. For instance, a polysaccharide from *Lycium barbarum*, a medicinal food traditionally used to retard ageing and improve neuronal function, is shown to attenuate ultraviolet B-induced cell viability decrease and ROS level increase in human keratinocytes HaCaT cells by promoting the nuclear translocation of Nrf2 and the expression of Nrf2-dependent ARE target genes [90]. This protective effect of *L. barbarum* polysaccharide can be neutralized by siRNA-mediated Nrf2 silencing, indicating an involvement of Nrf2/ARE pathway in the antioxidant effect of the polysaccharide [90]. Intriguingly, however, the above-mentioned polysaccharide DiPS is shown to increase oxidative survival through promoting nuclear translocation of transcription factor DAF-16/FOXO transcription factor but not SKN-1 (worm homologue of Nrf2) in wild-type *C. elegans* under paraquat exposure, demonstrating the antioxidant activity of the polysaccharide is associated with IIS, an evolutionarily conserved pathway that regulates organismal metabolism and lifespan, as DAF-16 is a key regulator in IIS [58].

Several signaling pathways related to cell death and survival are also involved in the antioxidant effect of food-derived polysaccharides. For example, hydrogen peroxide can induce apoptosis of rat pheochromocytoma PC12 cells via activation of p38 MAPK, while a polysaccharide from the fruiting bodies of the edible mushroom *Morchella importuna* increases the viability of hydrogen peroxide-exposed PC12 cells by inhibiting p38 MAPK phosphorylation [91]. In addition, hydrogen peroxide can inhibit the activation of PI3K/Akt signaling in human neuroblastoma SH-SY5Y cells, while sulfated polysaccharides prepared from fucoidan are able to increase the phosphorylation of PI3K/Akt and inhibit cell apoptosis [92]. Interestingly, the PI3K inhibitor LY294002 can partially prevent the beneficial role of the polysaccharide, demonstrating that modulation of PI3K/Akt pathway contributes to the protective effect of the sulfated polysaccharides against hydrogen peroxide cytotoxicity [92].

Recent studies provide clear evidence for the protective effects of food-derived polysaccharides against oxidative stress. Many polysaccharides exhibit potent reducing power, total antioxidant capacity and scavenging ability against free radicals in vitro. Moreover, some polysaccharides can decrease ROS and peroxidation product levels, improve antioxidant defense system and regulate stress-related signaling events to attenuate oxidative damage in cellular and animal models exposed to a variety of external stimuli, such as hydrogen peroxide, paraquat, ultraviolet radiation and virus. Together, these findings suggest a potential of these dietary polysaccharides to maintain health and prevent oxidative stress-related disorders.

## 3. Alleviation of Neurodegeneration by Food-Derived Antioxidant Polysaccharides

It is known that oxidative stress and chronic inflammation are two intertwined pathological events in NDD [85]. Excessive ROS can modulate inflammatory signaling to up-regulate the expression of pro-inflammatory factors such as cytokines, which act as potent stimuli in brain inflammation [93,94]. In turn, elevated inflammatory stress further provokes ROS generation via multiple pathways such as nuclear factor κB (NF-κB) signaling [85]. On the other hand, abnormal protein aggregation is known to be a common pathological hallmark of late-onset NDD. These protein aggregates, including amyloid-β peptide (Aβ) aggregates in AD and polyQ aggregates in HD, can induce neuronal damages through induction of oxidative stress, inflammation and mitochondrial dysfunction [95,96,97]. Oxidative stress can also promote the aggregation of pathogenic proteins as ROS modified-proteins tend to form aggregates [98]. In addition, a variety of chemical interventions, including excitatory amino acids such as glutamate, *N*-methyl-d-aspartate (NMDA) and kainic acid; neurotoxins such as 1-methyl-4-phenyl-1,2,3,6-tetrahydropyridine (MPTP) and 6-hydroxydopamine (6-OHDA); and ageing-promoting agents such as d-galactose, are also shown to induce neurodegenerative symptoms via oxidative and inflammatory stresses [83,99,100]. As oxidative stress plays a pivotal role in neurodegeneration, antioxidant strategies, including food-derived antioxidant polysaccharides, are shown to attenuate neuronal damage and improve cognitive and motor functions in a range of neurodegenerative models (Table 2) [101,102,103,104,105,106,107,108,109,110,111,112,113,114,115,116,117,118,119,120,121,122,123,124,125,126,127,128,129,130,131,132,133,134,135,136,137,138,139,140,141,142].

### 3.1. Effects on Alzheimer’s Disease

AD is characterized by amyloid plaques and neurofibrillary tangles in the brain, which lead to progressive memory loss and cognitive decline [143]. As global population ages, AD has become a major public health concern. Among therapeutic and nutritional interventions, growing evidence has shown that adequate intake of antioxidants may be helpful to reduce neuronal damages and alleviate AD symptoms [144,145]. For example, dietary intake of α-tocopherol or combined tocopherols shows beneficial effects to alleviate age-related cognitive decline and lower AD risk [146,147].

Antioxidant polysaccharides from various food sources are also found to inhibit Aβ-mediated neurotoxicity in experimental models (Table 2). The polysaccharide PS-WNP from the medicinal food *Polygonatum sibiricum*, for instance, is shown to significantly attenuate Aβ-induced apoptosis of PC12 cells by alleviating mitochondrial dysfunction, regulating apoptosis-related protein Bax and Bcl-2 levels, inhibiting apoptotic executor caspase-3 activation and enhancing Akt phosphorylation [105]. In rats injected with Aβ40 aggregates, fucoidan is shown to attenuate learning and memory deficits by elevating SOD and GPx activities and decreasing MDA content, Bax/Bcl-2 ratio and caspase-3 activity in hippocampal tissue [104]. Using transgenic *C. elegans* models that overexpress Aβ proteins, the *D. indusiata* polysaccharide DiPS is shown to alleviate chemosensory behavior dysfunction, which is associated with reduction of ROS level and MDA content, increase of SOD activity and alleviation of mitochondrial dysfunction [58]. Antioxidant polysaccharides are also shown to modulate pathogenic protein aggregation, e.g., *L. barbarum* polysaccharides can reduce Aβ42 protein level in hippocampal tissue and improve the performance of learning and memory in APP/PS1 mice [109]. Intriguingly, *L. barbarum* polysaccharide is also shown to inhibit the apoptosis and reduce cleaved-tau protein level, the main component of neurofibrillary tangles in AD patients, in rat primary cortical cells exposed to homocysteine, a sulfur-containing amino acid associated with several NDD [128]. Moreover, several studies have uncovered that the regulatory effect of polysaccharides on protein aggregation is through the interaction with aggregation-prone proteins, and this effect is influenced by the chemical structure of polysaccharides. For example, four glycosaminoglycans from different animal tissues are shown to inhibit the neurotoxicity of serum amyloid P component and its interaction with Aβ, and the inhibitory efficacy is correlated with the uronic acid content in glycosaminoglycans [148]. In addition, the well-known glycosaminoglycan heparin is reported to bind with Aβ and promote amyloid fibrillogenesis, while low molecular weight heparin can prevent Aβ aggregation by blocking β-sheet formation and inhibiting fibril formation, suggesting that the molecular weight of polysaccharides may also affect their interaction with proteins [149,150]. Together, these studies demonstrate that the neuroprotective effects of food-derived polysaccharides in AD-like models correlate with their modulation of oxidative and related stresses.

### 3.2. Effects on Parkinson’s Disease

PD is a chronic and progressive NDD characterized by selective loss of dopaminergic neurons in the substantia nigra pars compacta and abnormal accumulation of Lewy bodies in these neurons [151]. The major clinical symptoms of PD include motor symptoms such as tremor and bradykinesia, and neuropsychiatric symptoms such as cognitive decline and anxiety [152]. Current clinic therapy for PD only concentrates on symptomatic management as the available therapeutics do not prevent disease progression [153].

Recent studies have shown that several food-derived antioxidant polysaccharides are capable of inhibiting the neurotoxicity mediated by MPTP and 6-OHDA, which can selectively induce dopaminergic neuron death and cause PD-like motor deficits in experimental models (Table 2). For instance, the polysaccharides from the seaweed *Saccharina japonica* and from the sea cucumber *Stichopus japonicus* can increase 6-OHDA-induced reduction of cell viability in SH-SY5Y cells and murine embryonic stem MES 23.5 cells, respectively [119,142]. The *S. japonicus* polysaccharides are shown to increase SOD activity, regulate the level of apoptosis-related proteins, inhibit NF-κB and p38 MAPK activation and activate PI3K/Akt pathway, indicating the involvement of antioxidant, anti-apoptotic and anti-inflammatory signaling pathways in its neuroprotective effect [119]. Using MPTP-injected mouse models, low molecular weight fucoidan DF and its two fractions DF1 and DF2 are shown to ameliorate dopaminergic neuron injury and prevent dopamine depletion in the substantia nigra through enhancing antioxidant enzyme activities and inhibiting neuronal apoptosis [154]. Interestingly, DF1 exerts better neuroprotective activity than DF and DF2 in general, and their monosaccharide compositions are different: DF1 is a hetero-polysaccharide with low content of fucose and high content of uronic acid and other monosaccharides, while DF2 mainly consists of fucose and galactose, suggesting that chemical composition may play an important role in the neuroprotective activity of fucoidan [154]. In addition, a polysaccharide from the edible microalga *Chlorella pyrenoidosa* is recently shown to reduce bradykinesia, inhibit the loss of striatal dopamine and its metabolites, and increase tyrosine hydroxylase in MPTP-injected mice [117]. The polysaccharide can also elevate the levels of small intestinal secretory immunoglobulin A, a protein that is crucial for the immune function of mucous membranes, in mice serum [117], and has been previously shown to enhance immune function [155]. As immune system dysfunction is known to contribute to PD development and progression [156], immune-related therapies may be a useful strategy to reduce disease risks and retard disease progression [157].

### 3.3. Effects on Huntington’s Disease

HD is an autosomal-dominant neurodegenerative disorder that is clinically manifested by a variety of motor, cognitive and psychiatric deficits [158]. This disease is caused by an abnormal expanded CAG trinucleotide repeat in the huntingtin gene on the short arm of chromosome 4. In normal individuals, the average number of CAG repeats in the huntingtin gene is 17–20; when the number of repeats exceeds 36, the risk of developing HD is significantly increased [159]. The prevalence of HD varies geographically, with the highest rates in Europe (~10–15 per 100,000 individuals) and lower rates in Asia and Africa [160]. Similar with AD and PD, currently there is no efficient treatment for HD.

Among various pharmacological interventions, natural antioxidants such as epigallocatechin gallate and salidroside have been found to alleviate HD-like symptoms in transgenic cellular and animal models [161,162]. Interestingly, several recent studies have uncovered that food-derived antioxidant polysaccharides also have beneficial effects in HD-like animal models (Table 2). For example, the *E. brevicornum* polysaccharide EbPS-A1 can alleviate polyQ-mediated chemosensory dysfunction in transgenic *C. elegans* model HA759 [38], which expresses a polyQ tract of 150 glutamine repeats in amphid sensilla (ASH) neurons, leading to progressive ASH death and chemotactic behavior deficit [163]. EbPS-A1 also reduces ROS level, inhibits lipid peroxidation and enhances antioxidant enzyme activities in HA759 nematodes, indicating that the antioxidant activity of the polysaccharide contributes to its protective effect against polyQ neurotoxicity [38]. Other studies suggest that some antioxidant polysaccharides exert their neuroprotective effects by targeting polyQ aggregate itself, e.g., *L. barbarum* polysaccharide not only increases the viability of HEK293 cells that express mutant-huntingtin containing 160 glutamine repeats but also improves motor behavior and lifespan in HD-related transgenic mice [122]. The neuroprotective effect of *L. barbarum* polysaccharide against mutant-huntingtin toxicity in both cellular and mouse models are shown to be associated with reducing mutant-huntingtin levels and activating AKT [122]. These studies provide an important insight into the therapeutic potential of food-derived antioxidant polysaccharides in HD.

### 3.4. Effects on Other Neurodegenerative Symptoms

Several recent studies have shown that food-derived antioxidant polysaccharides are capable of inhibiting excitatory amino acid-mediated neurotoxicity, which is implicated in many NDD [99,164]. For instance, *L. barbarum* polysaccharide can increase cell viability and suppress JNK activation in glutamate-exposed rat primary cortical neurons [129], suggesting an involvement of MAPK pathway in the neuroprotective effect of the polysaccharide. Another example is *G. lucidum* polysaccharide, which is shown to alleviate epileptic symptoms and up-regulate the expression of calcium/calmodulin-dependent protein kinase II, a kinase that plays an important role in calcium transfer in neurons, in kainic acid-injected rats [123]. As calcium overload mediates excitatory amino acid-induced neurotoxicity [164], prevention of calcium transporting may contribute to this neuroprotective effect of *G. lucidum* polysaccharide. In addition, other chemicals can also induce cognitive impairment and behavior deficit through increase of oxidative and inflammatory stresses, and the polysaccharides isolated form mushrooms, medicinal herbs and algae are reported to attenuate neurodegenerative symptoms induced by these toxic chemicals. For instance, a polysaccharide from *Pleurotus ostreatus* can decrease escape latency in Morris water maze test and increase passive avoidance latency in step-down test in rats under d-galactose and aluminum chloride challenge [125]. *P. ostreatus* polysaccharide also reduces MDA level and elevates SOD, GPx and CAT activities [125], indicating that the behavior-improving capability of the polysaccharide correlates with reduction of oxidative stress.

**Figure 1:**
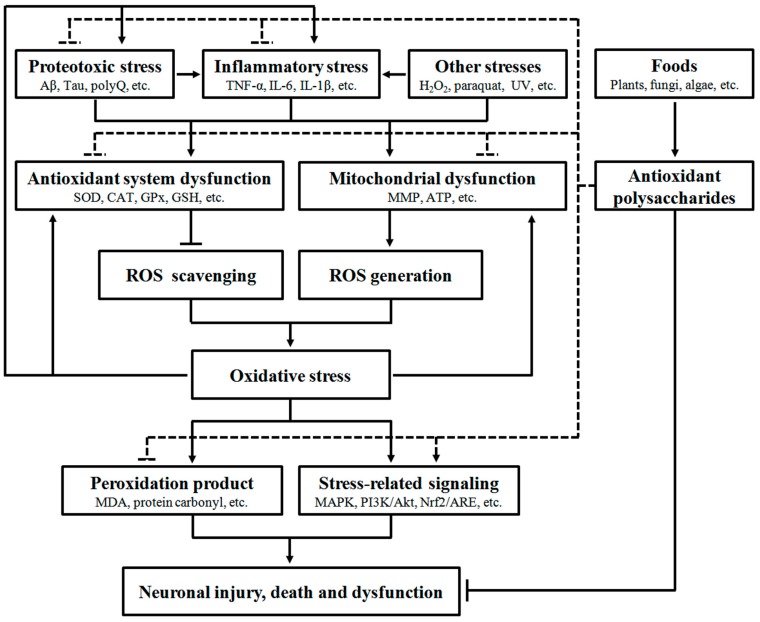
Pharmacological intervention of neurodegeneration by food-derived antioxidant polysaccharides. A number of extrinsic and intrinsic stresses such as proteotoxic stress, inflammatory stress and chemical interruption can stimulate oxidative stress through impairing the function of antioxidant system and mitochondria. Increase of oxidative stress can promote pathogenic protein aggregation and inflammation, eventually leading to neuronal injury, death and dysfunction via multiple biochemical pathways (solid line). However, food-derived antioxidant polysaccharides can exert beneficial effects to suppress neurodegeneration via attenuating oxidative, inflammatory and proteotoxic stresses and regulating stress-related signaling (dashed line).

A large body of evidence has confirmed that oxidative stress can interact with many other stresses to induce neurodegeneration, indicating its significant role in NDD development. Food-derived antioxidant polysaccharides are recently shown to alleviate neuronal injury, death and dysfunction through modulation of multiple oxidative stress-related pathways, including antioxidant defense system, mitochondrial function, peroxidation products, protein aggregation, inflammation and stress-related signaling (Figure 1), demonstrating their pharmacological potentials in NDD.

## 4. Conclusions

Food-derived polysaccharides have been shown to scavenge free radicals in vitro and reduce oxidative damages in cellular and animal models, and their in vivo antioxidant capacities are related with regulation of peroxidation products, antioxidant defense system and stress-related signaling. As oxidative stress is closely associated with neurodegeneration, some antioxidant polysaccharides are also tested for their anti-NDD activity and found to attenuate neuronal damages and dysfunction in a number of neurodegenerative models. The neuroprotective effects of polysaccharide are associated with alleviation of multiple stresses, including oxidative, inflammatory and proteotoxic stresses (Figure 1). Therefore, consumption of foods rich in antioxidant polysaccharides may not only reduce oxidative damage but also provide protection against oxidative stress-related disorders. It is noted that most recent studies focus on the antioxidant polysaccharides from terrestrial plants and fungi, and relatively less attention is paid to marine organisms although they represent a rich resource of bioactive polysaccharides. In addition, many food-derived antioxidant polysaccharides are shown to have potent immunomodulatory effects, and therefore it would be interesting to explore the involvement of immunomodulation in the neuroprotective effect of antioxidant polysaccharides.

## Figures and Tables

**Table 1:** The antioxidant activities and mechanisms of food-derived polysaccharides.

Source	Polysaccharide	Test Model	Protective Effect	Potential Mechanism	Ref.
**Vegetables**					
*Zizania latifolia*	ZLPs-W	In vitro assays	Scavenging activity against DPPH and ·OH		[24]
*Daucus carota*	CWSP	In vitro assays	Scavenging activity against DPPH, reducing power, prevention of β-carotene bleaching	Ferrous chelating ability	[25]
*Cucurbita maxima* Duchesne	WSP	In vitro assays	Scavenging activity against DPPH, inhibition of ascorbic acid oxidation	SOD-like activity	[26]
*Solanum tuberosum*	PPPWs	In vitro assays	Scavenging activity against DPPH and ABTS, reducing power, total antioxidant capacity		[27]
*Potentilla anserine*	PAP	H_2_O_2_-exposed murine splenic lymphocytes	Apoptosis rate↓		[28]
*Psidium guajava*	PS-PGL	In vitro assays; H_2_O_2_-exposed Vero cells and zebrafish	Scavenging activity against DPPH, ·OH and alkyl radicals in vitro; Cell viability↑, DNA fragmentation↓, nuclear condensation and morphological disruption↓ in Vero cells; Survival↑, heart-beating rate↓, cell death↓ in zebrafish embryos	ROS level↓ in Vero cells; ROS level↓, MDA content↓ in zebrafish embryos	[29]
**Fruits**					
*Malus pumila*	APPS	In vitro assays	Scavenging activity against DPPH, O_2_^−^· and ·OH, reducing power		[30]
*Diospyros kaki* L.	PFP	In vitro assays	Scavenging activity against DPPH, O_2_^−^· and ·OH, reducing power		[31]
Seed watermelon	SWP	H_2_O_2_-exposed PC12 cells	Cell viability↑, LDH release↓	ROS level↓, 8-OHdG content↓, caspase-3 and caspase-9 activities↓, MMP↑	[32]
**Cereals and Beans**					
Rice bran	RBP2	In vitro assays	Scavenging activity against DPPH, O_2_^−^·, ·OH and ABTS, reducing power	Ferrous chelating ability	[33]
Wheat bran	Feruloyl oligosaccharides	AAPH-exposed human erythrocytes	Erythrocyte hemolysis↓	GSH level↓, MDA content↓, PCG level↓	[34]
*Glycine max* (L.) Merr.	MSF	In vitro assays	Scavenging activity against ABTS, reducing power		[35]
*Cicer arietinum* L. hull	CHPS	In vitro assays; H_2_O_2_-exposed PC12 cells	Scavenging activity against ABTS, DPPH O_2_^−^; reducing power in vitro; Cell viability↑		[36]
**Herbs**					
*Dioscorea opposita*	Yam polysaccharide	In vitro assays	Scavenging activity against O_2_^−^· and ·OH		[37]
*Epimedium brevicornum* Maxim.	EbPS-A1	In vitro assays; PQ-exposed *C. elegans*	Scavenging activity against DPPH and ·OH in vitro; Survival rate↑ in *C. elegans*	ROS level↓, MDA content↓, SOD and CAT activities↑ in *C. elegans*	[38]
*Chuanminshen violaceum*	CVPS	In vitro assays; d-Gal-treated ICR mice	Scavenging activity against DPPH, O_2_^−^· and ·OH in vitro; Body weights and spleen indices↑ in mice	Activities and mRNA levels of Mn-SOD, Cu/Zn-SOD, GPx and CAT↑, MDA content↓ in mouse liver, heart and brain	[39]
*Radix Rehmanniae*	RRPs	UV-irradiated mice		GSH level↑, SOD, CAT and GPx activities↑, MDA content↓, IL-2, IL-4 and IL-10 levels↑	[40]
*Lycium barbarum*	LBPs	H_2_O_2_-exposed SRA01/04 cells	Cell viability↑, apoptotic rate↓, ratio of ageing cells↓, G0/G1 cell cycle phase arrest↓	ROS level↓, MMP↑, Bcl-2 protein level↑, Bax protein level↓, MDA content↓, SOD activity↑, GSH level↑	[41]
*Angelica sinensis*	ASP	H_2_O_2_-exposed PC12 cells; SD rats with middle cerebral artery occlusion	Cell viability↑, apoptosis rate↓ in PC12 cells; Number of microvessels in rat brain↑	ROS level↓, MMP↑ in PC12 cells; SOD and GPx activities↑ in rat cortex	[42]
*Sophora subprosrate*	SSP	PCV-2 infection RAW264.7 cells		Activities of Total-SOD, Cu/Zn-SOD and Mn-SOD↑, mRNA levels of Mn-SOD↑ and NOX2↓, NOX2 protein level↓, MMP↑	[43]
*Cynomorium songaricum* Rupr.	CSP	H_2_O_2_-exposed PC12 cells	Cell viability↑, ratio of sub G1and S phase↓, ratio of G2/M phase↑, apoptosis rate↓, LDH release↓	ROS level↓,MDA content↓, 8-OHdG content↓, SOD and GPx activities↑, capase-3 and capase-9 activities↓	[44]
**Tea**					
Black tea	BTPS	In vitro assays	Scavenging activity against DPPH and ·OH		[45]
Green tea	TPS1	In vitro assays	Scavenging activity against DPPH, O_2_^−^· and ·OH, ferrous chelating ability, reducing power, total antioxidant capacity, inhibition of lipid hydroperoxide		[46]
*Gynostemma pentaphyllum* Makino	GPMMP	Cyclophosphamide-treated C57BL/6 mice	Spleen and thymus indices↑, CD4+ T lymphocyte counts↑, total antioxidant capacity↑	CAT, SOD and GPx activities↑, MDA content↓, GSH level↑, IL-2 level in sera and spleen↑	[47]
**Nuts**					
*Juglans regia* L.	SJP	In vitro assays	Scavenging activity against DPPH, ·OH and ABTS, reducing power		[48]
*Ginkgo biloba* L.	GNP	In vitro assays; Hyperlipemia mice	Scavenging activity against DPPH, O_2_^−^· and ·OH in vitro	CAT, SOD and GPx activities↑, MDA content↓ in mouse serum and liver	[49]
Other Plants					
*Zizyphus jujuba* Mill	ZJPa	In vitro assays	Scavenging activity against O_2_^−^· and ·OH	Ferrous chelating ability	[50]
*Aloe barbadensis* Miller	GAPS-1 and SAPS-1	In vitro assays	Scavenging activity against O_2_^−^·, ·OH and H_2_O_2_, reducing power, MDA content↓	Ferrous chelating ability	[51]
*Anoectochilus roxburghii*	ARPT	CCl_4_-treated Kunming mice	Hepatocyte necrosis↓, serum alanine transaminase and aspartate transaminase activities↓	MDA level↓, SOD, CAT and GPx activities↑, GSH level↓, mRNA levels of TNF-α, IL-6 and Bax↓, protein levels of TNF-α, IL-6, NF-κB and cleaved-caspase 3↓ in liver	[52]
*Opuntia dillenii* Haw	CP	H_2_O_2_-exposed PC12 cells	Cell viability↑, LDH release↓, apoptosis rate↓	ROS level↓, ratio of Bax/Bcl-2 mRNA level↑	[53]
*Camellia oleifera* Abel	SCP1	In vitro assays; PQ-exposed *C. elegans*	Scavenging activity against O_2_^−^· and ·OH in vitro; Survival rate↑ in *C. elegans*	Ferric chelating ability in vitro; SOD, CAT and GPx activities↑, MDA content↓ in *C. elegans*	[54]
*Taraxacum officinale*	TOP2	LPS or *t*-BHP-exposed RAW 264.7 cells	NO production↓ in LPS-exposed cells; Cell viability↑ in *t*-BHP-exposed cells	Protein levels of TNF-α, p-IκBα, p-p65, p-Akt, iNOS and heme oxygenase 1↓	[55]
**Mushrooms**					
*Ganoderma lucidum*	*G. lucidum* polysaccharide	Isoproterenol-treated albino rats	Creatinine kinase and LDH activities↓ in serum, cardiac muscle fibers with mild hyalinization	ROS level↓, MDA content↓, SOD and GPx activities↑, GSH level↑, activities of Krebs cycle dehydrogenases and mitochondrial complexes↑, MMP↑	[56]
*Lentinus edodes*, *Ganoderma applanatum*, *Trametes versicolor*	Mushroom polysaccharides	In vitro assays	Scavenging activity against DPPH, reducing power, inhibition of linoleic acid peroxidation	Ferric chelating ability	[57]
*Dictyophora indusiata*	DiPS	PQ-exposed *C. elegans*	Survival rate↑	ROS level↓, SOD activity↑, MDA content↓, MMP↑, ATP content↑, DAF-16 activation↑	[58]
**Other Fungi**					
*Auricularia auricula*	AAP1	In vitro assays; PQ or H_2_O_2_-exposed *C. elegans*	Scavenging activity against DPPH, O_2_^−^· and ·OH, reducing power in vitro; Survival rate↑ in *C. elegans*	Ferric chelating ability in vitro; ROS level↓, SOD and CAT activities↑ in *C. elegans*	[59]
*Tremella fuciformis*	TP	UV-irradiated SD rats	Water and collagen content↑, glycosaminoglycan↓, endogenous collagen breakdown↓, ratio of type I/III collagen↑ in rat skin	SOD, GPx and CAT activities↑	[60]
**Algae**					
*Porphyra haitanesis*	*P. haitanesis* polysaccharide	In vitro assays; H_2_O_2_-exposed rat erythrocytes and liver microsome	Scavenging activity against O_2_^−^· and ·OH in vitro; Erythrocyte hemolysis↓; lipid peroxidation of rat liver microsome↓		[61]
*Laminaria japonica*	LJPA-P3	In vitro assays	Oxygen radical absorbance capacity, scavenging activity against ABTS		[62]
*Fucus vesiculosus*	*F. vesiculosus* polysaccharide	In vitro assays	Ferric reducing antioxidant power		[63]
*Ulva pertusa*	*U. pertusa* polysaccharide	In vitro assays	Scavenging activity against O_2_^−^· and ·OH, reducing power	Ferric chelating ability	[64]
Brown seaweed	Fucoidan	UV-irradiated HS68 cells		ROS level↓, MDA content↓, GSH level↑	[65]
*Nostoc commune*	*Nostoc* polysaccharide	In vitro assays; PQ-exposed *C. elegans*	Scavenging activity against O_2_^−^· and ·OH in vitro; Survival rate↑ in *C. elegans*	SOD, CAT and GPx activities↑, MDA content↓ in *C. elegans*	[66]
**Milkproducts**					
Milk fermented with lactic acid bacteria	Exopolysaccharides	UV-irradiated hairless mice	Erythema formation, dryness and epidermal proliferation, cyclobutane pyrimidine dimers↓ in mouse skin	mRNA levels of xeroderma pigmentosum complementation group A↑, ratio of mRNA levels of IL10/IL12α and IL10/IFN-γ↓ in mouse skin	[67]
**Wine**					
Red wine	PS-SI	In vitro assays	Scavenging activity against ·OH, oxygen radical absorbance capacity		[68]
**Probiotics**					
*Bifidobacterium animalis* RH	EPS	In vitro assays; d-Gal-treated Kunming mice	Inhibition of linoleic acid peroxidation, total antioxidant capacity, scavenging activity against DPPH, O_2_^−^· and ·OH in vitro	Total antioxidant capacity, SOD, CAT and GPx activities↑, MDA content↓ in serum, GST activity and MDA content↓ in liver, MAO activity and lipofuscin level↓ in brain	[69]
*Bifidobacterium bifidum* WBIN03, *Lactobacillus plantarum* R31	B-EPS and L-EPS	In vitro assays; H_2_O_2_-exposed rat erythrocytes	Scavenging activity against DPPH, O_2_^−^· and ·OH, inhibition of lipid peroxidation in vitro; Erythrocyte hemolysis↓		[70]
**Meat**					
*Haliotis discus hannai* Ino	ASP-1	In vitro assays	Scavenging activity against O_2_^−^·		[71]
*Crassostrea hongkongensis*	CHPs	In vitro assays	Scavenging activity against DPPH, ·OH and ABTS, inhibition of linoleic acid peroxidation		[72]
*Mytilus coruscus*	MP-I	CCl_4_-treated Kunming mice	Serum alanine transaminase and aspartate transaminase levels↓, necrosis of liver cells↓, immigration of inflammatory cells↓	MDA content↓, SOD activity↑ in liver	[73]

AAPH, 2,2′-Azobis(2-amidinopropane) dihydrochloride; ABTS, 2,2′-Azino-bis(3-ethylbenzothiazoline-6-sulfonic acid); CAT, catalase; CCl_4_, carbon tetrachloride; Cu/Zn-SOD, copper-zinc superoxide dismutase; d-Gal, d-galactose; DPPH, 2,2-diphenyl-1-picrylhydrazyl radical; GPx, glutathione peroxidase; GSH, glutathione; GST, glutathione S-transferase; H_2_O_2_, hydrogen peroxide; HS68 cells, human foreskin fibroblast line; IFN-γ, interferon-γ; IκBα, NF-κB inhibitor α; ILs, interleukins; iNOS, inducible nitric oxide synthase; LDH, lactate dehydrogenases; LPS, lipopolysaccharide; MAO, monoamine oxidase; MDA, malondialdehyde; MMP, mitochondrial membrane potential; Mn-SOD, manganese superoxide dismutase; NF-κB, nuclear factor-κB; NO, nitric oxide; NOX2, cytochrome b-245β chain; O_2_^−^·, superoxide anion; ·OH, hydroxyl radical; RAW 264.7 cells, murine macrophage cell line; PC12 cells, rat pheochromocytoma cell line; PCG, protein carbonyl group; PCV-2, porcine circovirus type 2; PQ, paraquat; ROS, reactive oxygen species; SOD, superoxide dismutase; SRA01/04 cells, SV40 T-antigen-transformed human lens epithelial cell line; *t*-BHP, tert-Butyl hydroperoxide; TNF-α, tumor necrosis factor α; UV, ultraviolet; 8-OHdG, 8-hydroxy-2’-deoxyguanosine.

**Table 2:** Protective effects and mechanisms of food-derived antioxidant polysaccharides in neurodegeneration models.

Source	Polysaccharide	Test Model	Protective Effect	Potential Mechanism	Ref.
*Ganoderma lucidum*	GLP	APP/PS1 transgenic mice	Learning and memory in MWM↑, neural progenitor cell proliferation↑	Aβ deposits↓, protein levels of p-FGFR1, p-ERK and p-Akt↑	[101]
Marine red algae	KCP	Aβ(25–35)-exposed SH-SY5Y cells	Cell viability↑, apoptosis rate↓	Protein level of cleavage caspase 3↓, JNK signaling activation↓	[102]
*Undaria pinnatifida* sporophylls	Fucoidan	Aβ(25–35) and d-Gal-exposed PC12 cells; d-Gal treated ICR mice	Cell viability↑, apoptosis rate↓ in PC12 cells; Learning and memory in MWM↑	Protein levels of cleaved caspase-3, caspase-8 and caspase-9↓, cytochrome c release↓, SOD activity↑, GSH level↑ in PC12 cells; Aβ deposits in hippocampus↓, SOD activity and GSH level↑ in serum, Ach content↑, ChAT activity↑ and AChE activity↓ in brain	[103]
*Laminaria japonica* Aresch.	Fucoidan	Aβ40-treated SD rats	Learning and memory in MWM, single-trial passive avoidance and eight-arm radial maze task↑	Ach content↑, ChAT activity↑, AChE activity↓, SOD and GPx activities↑, MDA content↓, Bax/Bcl-2 protein level ratio↓, cleaved caspase-3 protein level↓ in hippocampus	[104]
*Polygonatum sibiricum*	PS-WNP	Aβ(25–35)-exposed PC12 cells	Cell viability↑, apoptosis rate↓	Bax/Bcl-2 protein level ratio↓, MMP↑, cytochrome c release↓, cleaved caspase-3 protein level↓, caspase-3 activity↓, p-Akt protein level↑	[105]
*Lonicera japonica* Thunb.	LJW0F2	Aβ42-exposed SH-SY5Y cells	Cell viability↑	Aβ42 aggregates↓	[106]
*Echlonia Kurome* Okam.	AOSC	Aβ(25–35)-exposed SH-SY5Y cells	Cell viability↑, apoptosis rate↓, activation of astrocytes↓, cell redox activity↑	ROS level↓, TNF-α and IL-6 level↓, calcium influx in astrocytes↓	[107]
*Angelica sinensis*	AS	Aβ(25–35)-exposed Neuro 2A cells	Cell viability↑	ROS level↓, GSH level↑, MMP↑, mitochondria mass↑, TBARS content↓, autophagosomes or residual bodies↓	[108]
*Lycium barbarum*	*L. barbarum* polysaccharide	APP/PS1 transgenic mice	Learning and memory in MWM↑	Aβ deposits in hippocampus↓	[109]
*Lycium barbarum*	LBP-III	Aβ(25–35)-exposed rat primary cortical neurons	Maintain neurite fasciculation and neuron integrity	Caspase-3 and caspase-2 activities↓, p-PKR protein level↓	[110]
*Ganoderma lucidum*	GLA	Aβ(25–35)- or Aβ42-exposed rat primary cortical neurons	Apoptosis rate↓, synaptophysin immunoreactivity↑	DEVD-cleavage activity↓, protein levels of p-JNK, p-c-Jun, and p-p38↓	[111]
*Rubia cordifolia* L.	PS5	T-REx293 cells	Cell viability↑	Aβ42-EGFP aggregates↓	[112]
*Dictyophora indusiata*	DiPS	*C. elegans* CL2355	Survival rate↑, chemotaxis index↑	ROS level↓	[58]
*Gynostemma pentaphyllum* Makino	GPP1	Aβ(25–35)-exposed PC12 cells	Cell viability↑, LDH release↓, DNA fragmentation↓	ROS level↓, MDA content↓, SOD activity↑, GSH level↑, Calcium overload↓, MMP↑, Bcl-2 protein level↑, protein levels of Bax, cytochrome c and cleaved caspase-3↓	[113]
*Lycium barbarum* L.	LBP	6-OHDA-exposed PC12 cells	Cell viability↑, nuclear morphology changes↓, apoptosis rate↓	ROS and NO levels↓, calcium overload↓, protein-bound 3-nitrotyrosine level↓, protein levels of nNOS, iNOS and cleaved caspase-3↓	[114]
*Gynostemma pentaphyllum* Makino	GP	MPP^+^-exposed PC12 cells	Cell viability↑, LDH release↓, apoptosis rate↓	Cytochrome c release↓, caspase-3 and caspase-9 activities↓, Bax/Bcl-2 protein level ratio↓, protein levels of cleaved caspase-3 and poly (ADP-ribose) polymerase↓	[115]
*Spirulina platensis*	PSP	MPTP-treated C57BL/6J mice	Number of TH-immunoreactive neurons and DAT binding ratio in the substantia nigra pars compacta↑	TH and DAT mRNA levels in substantia nigra↑, SOD and GPx activity↑ in serum and midbrain	[116]
*Chlorella pyrenoidosa*	CPS	MPTP-treated C57BL/6J mice	Body weight↑, movement in pole test and gait test↑	Contents of DA, DOPAC and HVA↑, ratio of DOPAC and HVA to DA↓, TH mRNA level↑, striatal Emr1 mRNA level↓, TNF-α, IL-1β and IL-6 levels in serum↓, d-amino acid oxidase and secretory immunoglobulin A levels↑	[117]
*Gracilaria cornea* J. Agardh	SA-Gc	6-OHDA-treated Wistar rats	Locomotor performance in OFT, rotarod and apomorphine-induced rotation test↑, weight gain↑	DA and DOPAC content↑, NO_2_/NO_3_ and GSH level↑ in brain, p65, iNOS and IL1β mRNA levels↓, BDNF mRNA level↑	[118]
*Stichopus japonicus*	SJP	6-OHDA-exposed SH-SY5Y cells	Cell viability↑, apoptosis rate↓, LDH release↓	SOD activity↑, ROS level↓, NO release↓, MDA content↓, MMP↑, cytochrome c release↓, percentage of cells in S phase↑, Bax/Bcl-2 protein level ratio↓, protein levels of Cyclin D3, p-p53, p-p38, p-JNK1/2, p-p65, iNOS and p-IκB↓, cleaved caspase-9/caspase-9 and cleaved caspase-3/caspase-3 protein level ratio↓, p-Akt and IκB protein levels↑	[119]
*Hericium erinaceus*	EA	MPTP-treated C57BL/6 mice	Apoptosis rate↓, number of normal neurons↑, motor function in RT↑	Nitro-tyrosine and 4-HNE level↓, dopamine, NGF, and GSH level↑, protein levels of Fas, p-JNK1/2, p-p38, DNA damage inducible transcript 3, NF-κB and p65↓	[120]
*Epimedium brevicornum* Maxim.	EbPS-A1	*C. elegans* HA759	Avoidance index↑	ROS level↓, MDA content↓, SOD and CAT activities↑	[38]
*Turbinaria decurrens*	TD fucoidan	MPTP-treated C57BL/6 mice	Motor performance in OFT, Narrow beam walking and RT↑, nigral TH immunoreactivity↑	DA, DOPAC, and HVA content↑, TBARS level↓, GSH level↑, SOD and CAT activities↓, GPx activity↑, TH and DAT protein levels↑	[121]
*Lycium barbarum*	LBP	HEK293-160Q cells; HD-related transgenic mice	Cell viability↑ in HEK293 cells; Survival rate↑, weight gain↑, motor performance in RT↑ in mice	Soluble and aggregated huntingtin levels↓, caspase-3 activity↓, p-Akt/Akt and p-GSK3β/ GSK3β protein levels↑ in HEK293 cells; Mutant huntingtin level↓, p-Akt/Akt and p-GSK3β/ GSK3β protein levels↑ in mouse brain	[122]
*Ganoderma lucidum*	GLP	Kainic acid-treated Wistar rats	Frequency of epilepsy↓	CaMK II level↑, ERK1/2 level↓, calcium turnover↓, Caveolin-1 positive cells↑, NF-κB positive cells↓	[123]
*Hericium erinaceus*	HE	l-Glu-exposed PC12 cells; AlCl₃ and d-Gal-treated Balb/c mice	Differentiation rate↑, cell viability↑, apoptosis rate↓ in PC12 cells; learning, memory and locomotor in MWM, Autonomic activities and RT↑	β-tubulin III protein level↑, MMP↑, calcium overload↓, ROS level↓ in PC12 cells; Ach and ChAT contents in mouse serum and hypothalamus↑	[124]
*Pleurotus ostreatus*	POP	d-Gal and AlCl_3_-treated Wistar rats	Learning and memory in MWM and SDT↑, hippocampal impairment↓	AchE activity↓, in hippocampus, MDA content↓, SOD, GPx and CAT activities↑ in hippocampus, liver and serum, protein levels of APP, Aβ, BACE1 and p-tau↓, Protein phosphatase 2 protein level↑	[125]
*Sargassum fusiforme*	SFPS65A	SCO-, ethanol- and sodium nitrite-treated ICR mice	Learning and memory in SDT↑		[126]
*Sargassum fusiforme*	SFPS	d-Gal-treated ICR mice		CAT and SOD activities↑, MDA content in hearts and MAO in brains↓, protein levels of Nrf2, Bcl-2, p21 and JNK1/2↑, mRNA levels of Nrf2, Cu/Zn-SOD, Mn-SOD, glutamate cysteine ligase and GPX1↑, voltage dependent anion channel 1 protein level↓	[127]
*Lycium barbarum*	LBA	Homocysteine-exposed cortical neurons	Cell viability↑, apoptosis rate↓	LDH release and caspase-3 activity↓, p-tau-1 protein level↑, cleaved-tau protein level↓, p-ERK1/2 and p-JNK protein levels↓	[128]
*Lycium barbarum*	LBA	l-Glu- or NMDA-exposed cortical neurons	Cell viability↑, maintained their integrity and fasciculation of neurites	LDH release and caspase-3 activity↓, p-JNK-1/JNK protein level ratio↓	[129]
*Saccharomyces cerevisiae*	β-glucan	SCO-treated SD rats	Learning, memory, and locomotor in MWM and PTT↑	AChE activity↓	[130]
*Flammulina velutipes*	FVP	SCO-treated Wistar rats	Learning and memory in MWM and PTT↑	SOD and GPx activities↑, TBARS level↓, Ach, 5-HT, DA and NE content↑, ChAT activity↑, AChE activity↓, connexin 36 and p-CaMK II protein level↑ in hippocampus and cerebral cortex	[131]
*Lycium barbarum*	LBPs	SCO-treated SD rats	Learning and memory in MWM, NOR and OLR↑, cell proliferation and neuroblast differentiation in dentate gyrus↑	SOD and GPX activities↑, MDA content↓, Bax/Bcl-2 protein level ratio↓ in hippocampus	[132]
*Lycium barbarum*	LBP	d-Gal-treated Kunming mice	Weight gain↑, learning and memory in Jumping test↑, thymus and spleen indices↑	Lipid peroxidation, lipofuscin and MAO-B contents↓ in brain	[133]
*Polygonatum sibiricum*	PSP	SCO-treated Kunming mice	Learning and memory in SDT and Memory test↑	SOD and GPx activities↑, MDA content↓	[134]
*Panax ginseng*	WGOS	SCO-treated ICR mice	Learning and memory in MWM and NOR↑	mRNA levels of GFAP, IL-1β and IL-6↓ in hippocampus, number of GFAP-positive cells↓ in hippocampal subregions	[135]
*Lentinus edodes*	LT2	d-Gal-treated Kunming mice	Erythrocyte membrane fluidity↑	SOD and GPx activities↑ in liver, heart and brain	[136]
*Angelica* *sinensis*	ASP	d-Gal-treated C57BL/6J mice	Percentage of ageing cells↓	Advanced glycation end-product level in serum↓, ROS level↓, TAOC content↑, 8-OHDG content↓, 4-HNE level↓, protein levels of H2A histone family member X, p16, p21, p53, β-catenin, p-GSK-3β and transcription factor 4↓, mRNA levels of p16, p21 and β-catenin↓, GSK-3β protein level↑	[137]
*Tricholoma lobayense*	TLH-3	*t*-BHP-exposed HELF cells; d-Gal-treated Kunming mice	Cell viability↑, percentage of ageing cells↓, ratio of G0/G1phase↓, nucleic morphological changes↓ in HELF cells	ROS level↓, in HELF cells; SOD and CAT activities↑, MDA content↓, in mouse liver and serum	[138]
*Cuscuta chinensis* Lam	PCCL	d-Gal-treated SD rats	Apoptosis rate of cardiomyoctyes↓	Calcium overload↓, Bax/Bcl-2 protein level ratio↓, caspase-3 activity↓, cytochrome c release↓	[139]
*Ganoderma atrum*	PSG-1	d-Gal-treated Kunming mice	Weight gain↑, lymphocyte proliferation↑	MDA content↓, SOD, CAT and GPx activities↑, GSH level↑, GSSG level↓ in liver, brain and spleen	[140]
*Auricularia auricula-judae*	APP 1-a	d-Gal-treated Kunming mice	Spleen and thymus indexes↑	MDA content↓, SOD and GPx activities↑ in liver, serum and heart	[141]
*Saccharina japonica*	DJ0.5	6-OHDA-exposed MES 23.5 cells and SH-SY5Y cells	Cell viability↑		[142]

Aβ, amyloid-β peptide; Ach, acetylcholine; AChE, acetylcholinesterase; APP, amyloid precursor protein; BACE1, β-secretase 1; BDNF, brain-derived neurotrophic factor; CaMK II, calmodulin-dependent protein kinase II; CAT, catalase; ChAT, choline acetyltransferase; CL2355, a nematode that pan-neuronally expresses Aβ42; Cu/Zn-SOD, copper-zinc superoxide dismutase; d-Gal, d-galactose; DA, dopamine; DAT, dopamine transporter; DOPAC, 3,4-Dihydroxyphenylacetic acid; FGFR1, fibroblast growth factor receptor 1; GFAP, glial fibrillary acid protein; GPx, glutathione peroxidase; GSH, glutathione; GSK-3β, glycogen synthase kinase-3β; GSSG, glutathione disulfide; HA759, a nematode that expresses HtnQ150 in ASH neurons; HEK293 cells, human embryonic kidney cell line; HELF cells, human embryonic lung fibroblast line; HVA, homovanillic acid; IκB, NF-κB inhibitor; iNOS, inducible nitric oxide synthase; LDH, lactate dehydrogenases; l-Glu, l-glutamate; MAO, monoamine oxidase; MES 23.5 cells, rodent mesencephalic neuronal cell line; MDA, malondialdehyde; MMP, mitochondrial membrane potential; MPTP, 1-methyl-4-phenyl-1,2,3,6-tetrahydropyridine; MPP^+^, 1-methyl-4-phenylpyridinium; MWM, Morris water maze; NE, norepinephrine; Neuro 2A cells, murine neuroblastoma cell line; NMDA, *N*-methyl-d-aspartate; nNOS, neuronal nitric oxide synthase; NO, nitric oxide; NOR, novel object recognition; Nrf2, nuclear factor erythroid 2-related factor 2; OLR, object location recognition; OFT, open field test; PC12 cells, rat pheochromocytoma cell line; PS1, presenilin-1; PTT, probe trial test; ROS, reactive oxygen species; RT, Rotarod test; SCO, scopolamine; SDT, step-down test; SH-SY5Y, human neuroblastoma cell line; SOD, superoxide dismutase; TAOC, total antioxidant capacity; TBARS, thiobarbituric acid reactive substances; TH, tyrosine hydroxylase; TNF-α, tumor necrosis factor α; T-REx293, human embryonic kidney cell line transiently transfected with Aβ42-EGFP; *t*-BHP, tert-butylhydroperoxide; 4-HNE, 4-hydroxynonenal; 5-HT, 5-hydroxytryptamine; 6-OHDA, 6-hydroxydopamine; 8-OHDG, 8-hydroxydeoxyguanosine.
